# A web and telephone randomisation service in one hour – the benefits and challenges of delivering a configurable randomisation system

**DOI:** 10.1186/1745-6215-16-S2-P40

**Published:** 2015-11-16

**Authors:** John Turgoose, Will Crocombe

**Affiliations:** 1 University of Leeds, Leeds, UK

## 

No two trials are the same; even from an IS perspective. From differing data collection approaches, through to the myriad trial reports required to support them, we know there will always be a role for IT specialists delivering bespoke solutions to support the development and delivery of clinical trials.

When however in 2013 we focused on how we developed our randomisation services, we found that in the majority of cases they used a method of randomisation that could be broadly categorised into either minimisation or randomised permuted blocks. What we also knew was that we were spending a great deal of time and effort both developing and importantly validating these applications. In early 2014 this awareness combined with a growing clamour from our trial teams to deliver randomisation services over the web (previously we only supported an automated telephone system) drove us to rethink entirely our strategy for delivering randomisation. Could we build a single configurable system to meet the similar needs of our trial teams which still had the flexibility to deal with the unexpected? Could we harness a single configuration to deliver those services both over the web and the telephone? Could we harness this configurable system to reduce the development and validation burden, therefore allowing us to deliver these systems both more quickly and more efficiently?

